# Chromatin accessibility and transcriptome landscapes of *Monomorium pharaonis* brain

**DOI:** 10.1038/s41597-020-0556-x

**Published:** 2020-07-08

**Authors:** Mingyue Wang, Yang Liu, Tinggang Wen, Weiwei Liu, Qionghua Gao, Jie Zhao, Zijun Xiong, Zhifeng Wang, Wei Jiang, Yeya Yu, Liang Wu, Yue Yuan, Xiaoyu Wei, Jiangshan Xu, Mengnan Cheng, Pei Zhang, Panyi Li, Yong Hou, Huanming Yang, Guojie Zhang, Qiye Li, Chuanyu Liu, Longqi Liu

**Affiliations:** 1 BGI Education Center, University of Chinese Academy of Sciences, Shenzhen, 518083 China; 2 BGI-Shenzhen, Shenzhen, 518083 China; 3 China National Gene Bank, BGI-Shenzhen, Shenzhen, 518120 China; 4 State Key Laboratory of Genetic Resource and Evolution, Kunming Institution of Zoology, Chinese Academy of Science, Kunming, 650223 China; 5 Center for Excellence in Animal Evolution and Genetics, Chinese Academy of Science, Kunming, 650223 China; 6 BGI College, Zhengzhou University, Zhengzhou, 450000 China; 7 James D. Watson Institute of Genome Sciences, Hangzhou, 310013 China; 8 Section for Ecology and Evolution, Department of Biology, University of Copenhagen, Copenhagen, DK-2100 Denmark; 9 Shenzhen Bay Laboratory, Shenzhen, 518083 China

**Keywords:** Epigenomics, Transcriptomics

## Abstract

The emergence of social organization (eusociality) is a major event in insect evolution. Although previous studies have investigated the mechanisms underlying caste differentiation and social behavior of eusocial insects including ants and honeybees, the molecular circuits governing sociality in these insects remain obscure. In this study, we profiled the transcriptome and chromatin accessibility of brain tissues in three *Monomorium pharaonis* ant castes: queens (including mature and un-mated queens), males and workers. We provide a comprehensive dataset including 16 RNA-sequencing and 16 assay for transposase accessible chromatin (ATAC)-sequencing profiles. We also demonstrate strong reproducibility of the datasets and have identified specific genes and open chromatin regions in the genome that may be associated with the social function of these castes. Our data will be a valuable resource for further studies of insect behaviour, particularly the role of brain in the control of eusociality.

## Abstract (summary)

**Table:** 

Measurement(s)	mRNA • open_chromatin_region • brain
Technology Type(s)	RNA sequencing • ATAC-seq
Factor Type(s)	caste
Sample Characteristic - Organism	Monomorium pharaonis

Machine-accessible metadata file describing the reported data: 10.6084/m9.figshare.12111210

## Background & Summary

Eusocial insects have their societies based on caste polyphenism, where one or more queens are exclusively responsible for reproduction^1^. In contrast, workers, the largest population in the colony, are almost sterile and responsible for supporting the entire community through their labor, including collecting food, maintaining the nest and feeding/protecting the newly hatched larvae^2^. Eusociality in the hymenopteran insects has evolved 10 times independently^3,4^. Understanding eusociality in insects is important not only from an evolutionary or environmental perspective but also because it may provide clues into the behavior traits of higher species including humans.

Genes differentially expressed across castes in the brains of insects contribute to social behavior development^5,6^. Several studies have focused on the overlapping genes or pathways associated with the division of labor across different eusocial insect lineages and constructed a set of conserved gene regulatory networks^7,8^. In this regard, one of the key hypotheses for evolution of eusociality emphasized the important role of a core toolkit of genes involved in highly conserved pathways, such as metabolism and reproduction^9,10^. In addition, it is also widely accepted that certain single genes can play pivotal roles. For instance, increasing *insulin-like peptide 2* (*ilp2*) levels can break larval suppression and induce a stable division of labor in *Ooceraea biroi*^11^. Likewise, the neuropeptide corazonin inhibits the transition from worker to gamergate in *Harpegnathos saltator*^12^. Alternatively, many other studies have recognized the importance of taxonomically restricted genes in the evolution of eusocial behavior and performed a systematic comparison of the participation degree of shared genes and taxonomically restricted genes in eusocial division of labor^13–16^. Despite these relevant studies, the comprehensive lists of genes associated with eusocial behavior and their interrelationship are still unknown.

Besides gene expression, epigenetic regulation is also recognized as an important facet in the regulation of caste-specific behavior in insects. For example, histone modifications are critical regulators of caste determinations in *Camponotus floridanus*, as it was shown that distinct histone H3K27ac patterns exist between castes of *C. floridanus*^17^. Likewise, caste-specific behavior in *C. floridanus* can be reprogrammed by treatment with a small-molecular inhibitor of histone deacetylases, suggesting a regulatory role for histone acetylation in eusocial behavior plasticity^18^. The role of DNA methylation in caste determination has also been investigated in honeybees and, interestingly, some of the differentially methylated CpG sites correspond to regulatory regions of genes involved in metabolic pathways^19^. Additionally, distinct DNA methylation patterns in queen and worker larvae have been reported in another eusocial insect, the termite *Zootermopsis nevadensis*^19^.

Taken together, these reports suggested crucial roles of transcription and epigenetics in shaping caste differentiation and controlling social behavior in insects. However, a comprehensive dataset of both layers is still lacking, hampering further advances in the field of eusociality.

**Fig. 1:**
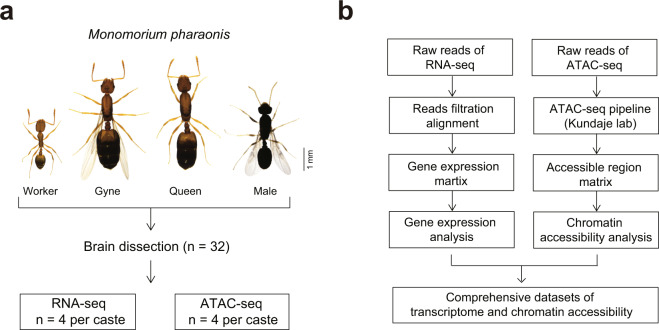
Overview of the experimental and data analysis workflow. (**a**) Four different adult groups from a *Monomorium pharaonis* colony were collected for RNA-sequencing and ATAC-sequencing profiling. (**b**) Analysis workflow for RNA-sequencing and ATAC-sequencing profiles.

Here, we constructed the transcriptome and chromatin accessibility landscapes of brain tissue of *Monomorium pharaonis* (Fig. 1a), which is the most ubiquitous house ant in the world^20,21^. *Monomorium pharaonis* consists of three adult castes, workers, queens, and males, with the queen caste containing unmated queens (gynes) and mature queens. These four adult groups possess distinct morphologies, lifespans and behaviors, making it an ideal model to explore the molecular and neural regulatory mechanisms of eusociality^22^. We sequenced 32 samples from the four groups of ants (16 RNA-seq and 16 ATAC-seq with four biological replicates per group). After data quality assessment and filtering, we obtained a total of 240 Gb high-quality base pairs for the RNA-sequencing, with more than 95% Q20 bases and approximately 149 million reads per sample. For the ATAC-sequencing, we obtained a total of 170 Gb high-quality base pairs reads, with approximately 106 million reads per sample.

## Methods

### Experimental design

Four adult groups (workers, gynes, males and queens) of *Monomorium pharaonis* were used for brain RNA-sequencing and ATAC-sequencing profiling. We collected eight brain samples per caste group to perform these assays. A total of 32 ant brains were used. Each brain was used as a biological sample for either the RNA-sequencing or ATAC-sequencing. The experimental design and analysis workflow are shown in Fig. 1.

### Animals

All procedures related to animals in this study were approved by the Institutional Review Board on Ethics Committee of BGI (Permit No. FT 19046). Two colonies of *Monomorium pharaonis* were created from a source colony (MP-MQ064) that was collected in June 2016 from Xishuangbanna in the Yunnan province in China. We pre-assigned about 200 workers, 10 queens and about 200 total larvae in each of the colonies. The age of the selected queens was unknown. The two colonies used in the study were created at the State Key Laboratory of Genetic Resource and Evolution, Kunming Institution of Zoology and then sent to the China National Gene Bank, BGI-Shenzhen. Ants were maintained for eight weeks before sampling, at a constant temperature of 25 °C and 50% humidity and fed with mealworm^12^. There were about 200 workers, 10 queens, 5 and 8 males, and 7 and 9 gynes in each colony, respectively, when sampling.

### Brain collection and RNA extraction

Designated ants were picked out and anaesthetized in a dissection dish on ice. The ants were then washed with ethanol and PBS twice, and dissected in PBS on ice under the light microscope (OLYMPUS, SZX16). To perform the dissection, a pair of forceps held the ant body while another pair of forceps inserted into the mouth gripped the head cuticle of the ant. The head was gently pulled off and the body discarded. Head cuticle was then gently peeled off with the forceps and the brain was removed. After carefully removing the surrounding trachea and ocelli, the ant brain was placed into PBS with 1 U/mL RNase inhibitor. All ants were dissected using the same method except that ocelli removal was not required for the workers. Brain samples were then washed twice with 500 μl PBS. All samples were collected during daytime (9:00 to 16:00). Whole-brain RNA was extracted immediately after dissection using an RNeasy Mini Kit (Qiagen) and eluted with 10 μl of nuclease-free water (NF-water, Ambion). The total amounts of RNA were measured using an RNA HS Qubit (Invitrogen).

### RNA-sequencing library construction

We applied an optimized Smart-seq2 method for RNA-sequencing library construction^23^. For cDNA generation, the following premixed reagent was added to each tube of RNA sample: 5 μl of 10 μM oligo-dT primer (5ʹ-AAGCAGTGGTATCAACGCAGAGTACT30VN-3ʹ, where “V” is either “A”, “C”, or “G”, and “N” is any base), 4.86 μl of 10 mM dNTP (New England Biolabs), 0.5 μl of 40 U/μl RNase inhibitor (New England Biolabs). Based on the amount of RNA, ERCC Spike-In (Ambion) was added to each tube. Then, the mix was incubated at 72 °C for 3 minutes and quickly placed on ice. Afterward, 20 μl of first-strand synthesis mix containing 8 μl of 5X first-strand buffer (Invitrogen), 2 μl of 100 mM dithiothreitol (DTT, Invitrogen), 2 μl of 200U/μl SuperScript II Reverse Transcriptase (Invitrogen), 8 μl of 5 M Betaine (Sigma), 0.24 μl of 1 M MgCl_2_ (Millipore), and 0.4 μl of 100 μM template switch oligo (5ʹ-AAGCAGTGGTATCAACGCAGAGTACATrGrG + G-3ʹ, where “r” indicates a ribonucleic acid base and “+” indicates a locked nucleic acid base, TSO, Exiqon) were added. RNA was reverse transcribed at 42 °C for 90 minutes, and 10 cycles of 50 °C for 2 minutes and 42 °C for 2 minutes and a final 70 °C for 5 minutes to inactivate the reverse transcriptase. cDNA amplification mix containing 50 μl of KAPA HiFi HotStart ReadyMix (KAPA Biosystems), 1 μl of 10 μM IS primer (5ʹ-AAGCAGTGGTATCAACGCAGAGT-3ʹ) and 9 μl of NF-water were then added. The amplification followed the following steps: 98 °C for 3 minutes, followed by 13 cycles of 98 °C for 20 seconds, 67 °C for 20 seconds, 72 °C for 6 minutes and finally 72 °C for 5 minutes. Afterwards, the PCR product was purified using 1X AMPure XP beads (Beckman Coulter). We measured cDNA concentration with the Qubit dsDNA HS Assay Kit 3.0 (Invitrogen) and analyzed size distribution on an HS DNA chip bioanalyzer (Agilent). Libraries were prepared using a fragmentation based method^24^. For each sample, 300 ng of cDNA was sheared with NEBNext dsDNA Fragmentase (New England Biolabs). Fragmented DNA was then purified, end-repaired, adapter-added, amplified and size-selected. Afterwards, the library size distribution was detected using an HS DNA chip bioanalyzer; the fragment length was in the range from 300 to 500 bp.

### ATAC-seq library preparation

We used a whole-brain transposition method for ATAC-sequencing library construction, as previously described^25^, with minor modifications. In brief, brains were dissected and washed twice with 500 μl ice-cold PBS. After centrifugation at 500 x g for 5 minutes, the samples were lysed with 50 μl lysis buffer (10 mM Tris-HCl, 10 mM NaCl, 3 mM MgCl_2_, 0.1% IGEPAL CA-630). We next mixed the samples harshly by pipetting and then centrifuged at 800 x g for 10 minutes. Supernatants were discarded and replaced with a 50 μl transposition reaction mix containing 10 mM TAPS-NaOH (pH 8.5), 5 mM MgCl_2_, 10% DMF, 2.5 μl of in-house Tn5 transposase (0.8 U/μl) and NF-water. This mixture was incubated at 37 °C for 30 minutes. Afterwards, transposed DNA was purified with MinElute Purification Kit (Qiagen) and amplified with primers containing barcodes.

### Sequencing

All data were generated with the BGISEQ-500 platform (MGI)^26^. First, the DNA concentration of each library was measured by Qubit dsDNA HS Assay Kit 3.0. A total of 300 ng of library DNA with different sample indexes was pooled for circular single-strand DNA (ssDNA circles). Then, ssDNA circles were used as a template to make DNA nanoballs by rolling circle replication. DNA nanoballs were loaded onto the sequencer flowcells for 100 bp paired-end for RNA-seq and 50 bp paired-end for ATAC-seq.

### RNA-sequencing dataset processing

**Fig. 2:**
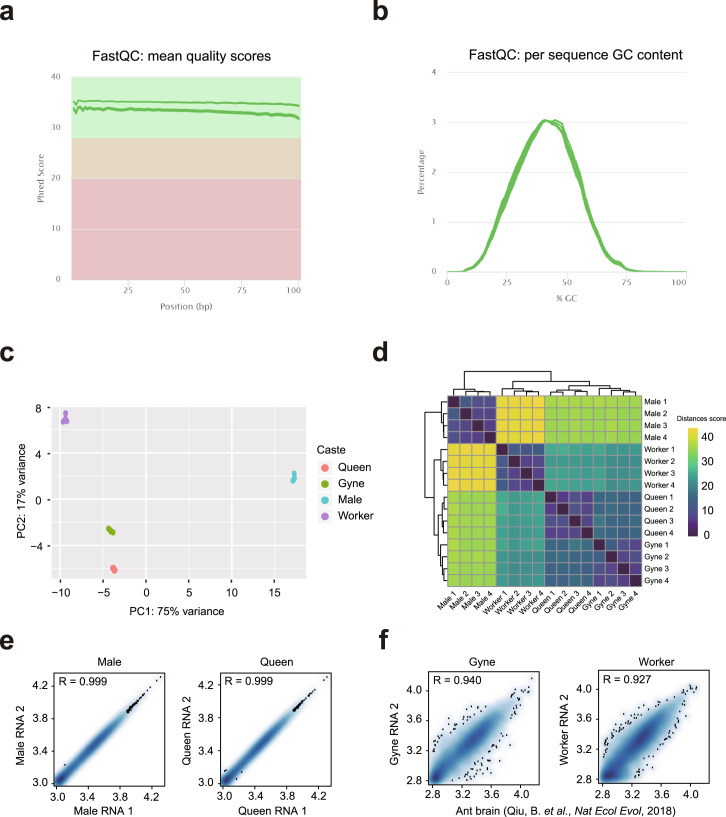
RNA-sequencing data quality metrics. (**a**) Mean quality values across each base position in the reads of RNA-sequencing datasets. (**b**) The GC content across the whole length of each sequence in read files of the RNA-sequencing datasets. (**c**) PCA plot of all 16 RNA-seq profiles. (**d**) Heatmap clustering of correlation coefficients across all 16 samples RNA-sequencing profiles. (**e**) Scatter plots showing the Pearson correlations between biological replicates. (**f**) Scatter plots showing the Pearson correlations between Qiu, B. *et al*. published datasets and our RNA-seq profiles.

Quality validation of raw reads was performed using FastQC (version 0.11.6)^27^. Reads of low quality were filtered using SOAPnuke (version 1.5.2)^28^. Adapter sequences, primers, poly-A tails were found and removed by cutadapt (version 1.16)^29^. Further quality control was performed by FastQC to ensure the cleaned data were suitable for downstream analyses. Quality control results^30^ were visualized using multiQC (version: 1.7)^31^. Statistical results of raw data and clean data are displayed in Table 1. Cleaned reads were mapped to the reference *Monomorium pharaonis* genome (GCA_003260585.2)^32^ using hisat2 (version 2.0.1-beta)^33^. The number of reads aligning to every gene of each sample were calculated with featureCounts (version 1.5.3)^34^ to generate a raw count matrix^30^. Aligned BAM reads were inputted into featureCounts (version 1.5.3) with a list of genomic features in Gene Transfer Format (GTF, ref_ASM326058v2_top_level.gff3.gz). To normalize read counts for sequencing depth and RNA composition, we used the median of ratios method in the R (version 3.5) package DESeq2 (version 1.5.3)^35^. The plotPCA function of DESeq2 (version 1.5.3) was used to assess the similarity of genomic specific gene expression patterns among different groups (Fig. 2c). Pearson correlation coefficients between samples (Fig. 2e, f) were calculated based on DEseq2 normalized data matrix.

**Table 1:** RNA-seq metadata and mapping statistics.

Sample ID	Number of raw reads	Number of clean reads	Percentage of clean reads	GC% (Clean reads)	Clean_Reads_Q20(%)	Number of mapped reads	Percentage of mapped reads
Gyne_RNA_1	119,235,814	102,841,766	86%	41%	95.80	67,659,598	65.79%
Gyne_RNA_2	172,470,928	146,346,992	85%	40%	95.89	99,164,722	67.76%
Gyne_RNA_3	203,319,094	171,549,152	84%	40%	95.98	116,893,592	68.14%
Gyne_RNA_4	181,881,936	154,635,550	85%	40%	95.54	103,327,476	66.82%
Male_RNA_1	222,617,504	166,697,332	75%	41%	96.41	107,553,118	64.52%
Male_RNA_2	171,733,948	134,612,370	78%	40%	95.57	85,721,158	63.68%
Male_RNA_3	163,818,754	130,940,972	80%	40%	95.33	84,941,408	64.87%
Male_RNA_4	194,226,758	152,877,424	79%	40%	95.65	100,715,648	65.88%
Queen_RNA_1	132,821,280	111,244,700	84%	41%	95.63	71,886,325	64.62%
Queen_RNA_2	172,918,396	141,405,076	82%	41%	95.75	93,129,383	65.86%
Queen_RNA_3	205,540,136	168,019,436	82%	40%	96.07	111,816,936	66.55%
Queen_RNA_4	211,761,094	173,207,908	82%	40%	96.16	116,828,734	67.45%
Worker_RNA_1	197,792,502	161,019,502	81%	41%	96.53	110,733,112	68.77%
Worker_RNA_2	182,845,616	152,743,314	84%	41%	96.04	102,536,588	67.13%
Worker_RNA_3	200,398,174	167,348,656	84%	40%	95.93	112,659,116	67.32%
Worker_RNA_4	187,128,572	158,590,210	85%	40%	95.39	106,556,762	67.19%

### ATAC-sequencing dataset processing

**Fig. 3:**
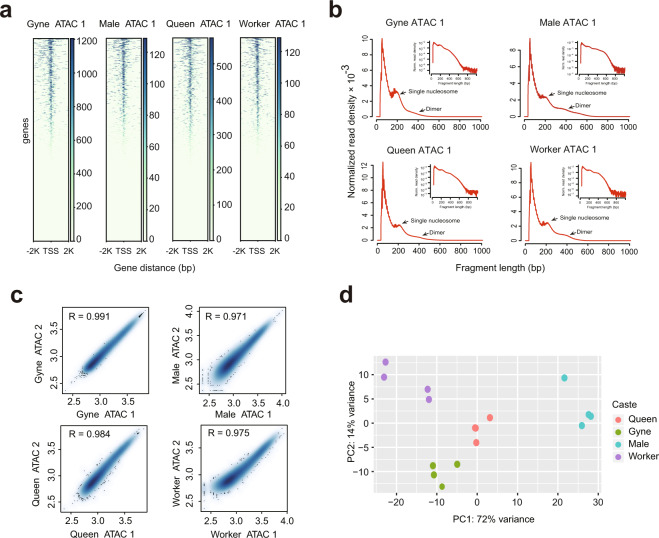
ATAC-sequencing data quality metrics. (**a**) The ATAC-sequencing signal enrichment around (2 K) the TSS for four representative samples (Gyne, Male, Queen, Worker). (**b**) Insert size distribution of ATAC-sequencing profiles for the same samples shown in 2a. (**c**) Scatter plots showing the Pearson correlations between biological replicates. (**d**) PCA plot of all 16 ATAC-sequencing profiles.

Raw ATAC-seqquencing data were processed including trimming, aligning, filtering, and quality controlling using an ATAC-sequencing pipeline^36^. MACS2 (version 2.1.2)^37^ based on python 2.7 was used to identify the peaks of accessible regions. We applied the IDR algorithm^38^ to identify peaks reproducible between replicates of each caste. Overlapping peaks were subsequently merged by bedtools (version: 2.26.0) intersect^39^ to produce the final consensus peak set. The full statistical results of data processing and the number of consensus peaks for each sample are listed in Table 2. A standard peak list was generated by merging peaks of all samples using bedtools merge^39^. The usable reads of each sample were then mapped to the regions of standard peaks using the intersect function of bedtools and the number of mapped reads was counted and listed in a matrix^30^. We normalized this raw count matrix using the median of ratios method of the R package DESeq2 (version 1.5.3). This normalized matrix was subjected to Pearson correlation coefficients calculation between replicates and principal component analysis (PCA) (Fig. 3c) by DEseq2.

**Table 2:** ATAC-seq metadata and mapping statistics.

Sample ID	Number of total reads	Number of mapped reads	Percentage of mapped reads	Number of usable reads	Percentage of usable reads	IDR peaks
Gyne_ATAC_1	172,172,820	163,448,845	94.93%	101,679,214	62.21%	38,585
Gyne_ATAC_2	137,815,754	130,970,332	95.03%	80,777,684	61.68%	38,585
Gyne_ATAC_3	198,553,750	189,962,529	95.67%	121,923,602	64.18%	38,585
Gyne_ATAC_4	124,501,796	115,458,488	92.74%	67,259,356	58.25%	38,585
Male_ATAC_1	48,790,218	42,106,287	86.30%	11,758,208	27.93%	16,685
Male_ATAC_2	53,764,102	47,327,040	88.03%	12,623,986	26.67%	16,685
Male_ATAC_3	45,813,866	40,610,146	88.64%	11,407,130	28.09%	16,685
Male_ATAC_4	42,554,344	38,399,918	90.24%	13,183,374	34.33%	16,685
Queen_ATAC_1	164,009,260	150,776,896	91.93%	59,420,000	39.41%	21,511
Queen_ATAC_2	91,740,402	82,496,005	89.92%	28,134,824	34.10%	21,511
Queen_ATAC_3	83,372,050	74,384,447	89.22%	20,508,800	27.57%	21,511
Queen_ATAC_4	175,570,098	163,084,283	92.89%	61,121,772	37.48%	21,511
Worker_ATAC_1	21,994,036	19,617,446	89.19%	8,703,090	44.36%	17,557
Worker_ATAC_2	83,557,276	77,937,726	93.27%	39,371,144	50.52%	17,557
Worker_ATAC_3	202,419,104	191,023,775	94.37%	122,065,556	63.90%	17,557
Worker_ATAC_4	57,754,220	53,530,040	92.69%	25,656,598	47.93%	17,557

### Identification of widely expressed or specific genes across the four groups

The raw gene expression matrix was normalized by Reads Per Million mapped reads (RPM). We calculated the average of RPM value, and the coefficient of variation (CV) between the four groups. We selected genes with mean value of RPM greater than 300 and the CV value less than 10% as co-expressed genes. We used the Shannon entropy^40^ to compute the specificity index for genes and we defined its relative gene expression level in a group type i as Ri = Ei/ΣE, where Ei is the RPM value for the gene in the group i, ΣE is the sum of RPM values in all groups and N is the total number of groups. The entropy score for each gene across groups was defined as H = −1 * sum (Ri * log2Ri) (1 < i < N), where the value of H ranges between 0 to log2(N). An entropy score close to zero indicates that the expression of the gene in question is highly specific based on the score distribution, whereas genes with entropy score less than 1.5 were selected as group-specific genes. This result was provided in Figshare^30^.

### Comparative analysis across groups

Comparative analysis was performed using DESeq2 R package. The fold change value between groups and the corresponding *P* value was calculated. We selected the genes or peaks with fold change ≥ 1 and Padj value ≤ 0.05 as differentially expressed genes (DEGs) or differentially accessible regions (DARs).

## Data Records

A complete list of the 32 ant brain samples is provided in Tables 1 and 2. All raw data in this study are available in the NCBI Gene Expression Omnibus (GEO)^41^ and in the CNGB Sequence Archive (CNSA)^42^ (https://db.cngb.org/cnsa/). The multiQC results and matrix of gene count and DEG statistics were submitted to Figshare^30^.

## Technical Validation

### RNA-sequencing metrics and reproducibility

A total of 16 RNA libraries were prepared and sequenced, with the sequencing depth ranging from 104.63 to 171.60 million reads. Raw reads were filtered, resulting in percentages of clean reads ranging between 75% and 86% (Table 1). The Q20 values for the clean reads were above 95% (Table 1). The quality of sequencing was validated by FastQC, then multiple results were compared with MultiQC and a representative result (all gyne samples) of the visualized Phred quality score per base was shown in Fig. 2a. The CG content ranged from 40% to 45%, following a normal distribution (Fig. 2b). Clean reads were then mapped to *Monomorium pharaonis* genome. A full statistics of quality control for each sample was displayed in Table 1.

The reproducibility of replicates of RNA-sequencing datasets was examined using PCA, in which samples were clearly separated by caste categories, with PC1 and PC2 jointly explaining 76% of the total variance in gene expression (Fig. 2c). Heatmap clustering of Pearson correlation coefficients from the comparison of the 16 datasets revealed a strong correlation between replicates of the same caste ants (Fig. 2d). Interestingly, three female groups (queens, gynes, and workers) had a nearer distance between each other than their distance to the male group. Pearson correlation analysis showed a correlation coefficient above 0.99 between replicates, revealing high reliability of the RNA-sequencing data (Fig. 2e). The RNA-sequencing data in our study were comparable with previously published RNA-sequencing data of gynes and workers^7^ (Fig. 2f). Taken together, these results suggest that our datasets are a reliable data resource for future studies.

### ATAC-sequencing quality control

We performed the quality assessment of ATAC-sequencing datasets by a variety of quality metrics (Table 2), including number of reads, mapping rate, and usable reads. Each sample obtained an average of 49 million usable reads after filtration, resulting in about 20, 000 reproducible peaks after IDR analysis (Table 2). We calculated the reads enrichment around transcription start sites (TSS) and observed a strong enrichment (Table 2 and Fig. 3a), suggesting the high quality of the datasets. This was also supported by the periodic pattern of fragment size, consistent with previous ATAC-sequencing profiles^43,44^ (Fig. 3b). Reproducibility between replicates was measured by Pearson correlation coefficients and all the replicates from each caste own the correlation coefficient more than 0.95 (Fig. 3c). The reproducibility of ATAC-sequencing datasets was further studied using PCA, where samples from the same caste tended to cluster together (Fig. 3d). As expected, we noted that the ATAC-sequencing samples presented a similar clustering result as RNA-sequencing, with the three female groups being closer to each other. Overall, these analyses demonstrated that our ATAC-sequencing datasets can reliably detect accessible regions in the genome and can be used to further explore the molecular foundation between epigenomic regulation and social behavior.

### Comparative analysis between castes

**Fig. 4:**
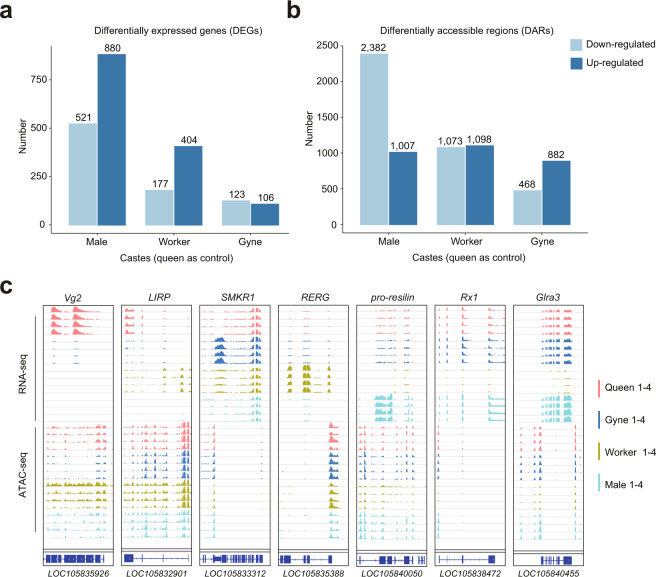
Identification of DEGs and chromatin accessible elements. (**a**) Histogram showing the number of DEGs (the queen is the control). (**b**) Histogram showing the number of DARs for the same groups shown in 4a. (**c**) Genome browser views of RNA-sequencing and ATAC-sequencing signals for the indicated genes and chromatin accessible-elements.

We identified a set of genes widely expressed in the brain of all castes and also caste brain-specific genes as well^30^. We found that genes co-expressed in the brains of four groups (600 genes) have a larger number than caste-specific genes (144 genes). These two sets of genes are provided in Figshare^30^, which can be used for further analysis and exploration. We counted the number of DEGs (Fig. 4a) or DARs (Fig. 4b) in gynes, workers and males compared with queens. We found that males show the biggest difference with queens in both gene expression and chromatin accessibility, suggesting that sex may be the most significant factor resulting in differential regulation of gene expression within the ant colony. On the contrary, gynes and queens presented the smallest difference, with only 229 DEGs and 1,350 DARs. The number of DEGs (583) and DARs (2,171) between queens and workers was almost twice as those between queens and gynes, suggesting higher similarity of the latter two.

We next investigated the relationship between expression and chromatin accessibility for DEGs across the four castes (Fig. 4c). Interestingly, we found that *locusta insulin-related peptide* (*LIRP*) and *vitellogenin-2* (*vg-2*) show high expression level in queens. LIRP is a type of 5 kDa peptide and first discovered from locust corpora cardiaca (CCs)-extracts^45,46^. *LIRP* contains 3 exons separated by 2 introns, resembling the vertebrate insulin genes^47,48^, whose function is to regulate eusocial division of labor and caste determination and was reported to show consistently higher expression in queens^9,11^. *Vitellogenin* (*Vg*) encodes for the major egg yolk protein precursor in insects and many other oviparous species^49^. Our finding is supported by a previous study demonstrating that *Vg* showed higher expression in reproductive groups of eusocial insects, as it functions as a lipid carrier that provisions developing oocytes with yolk and constitutes a reliable indicator of female reproductive activity^50^. *Small lysine-rich protein 1* (*SMKR1*), *ras-related and estrogen-regulated growth inhibitor* (*RERG*), and *pro-sesilin* were also identified as caste-specific genes expressed in gyne, worker and male brains, respectively. SMKR1 is a lysine-rich protein and may play an important role in brain development in unmated female ants^51^. RERG is a member of the RAS superfamily of GTPases and a estrogen-regulated growth inhibitor. The higher expression of *RERG* in worker is consistent with previous study of worker-biased genes in eusocial insects^52^. Resilin is an elastomeric protein found in many insects^53^. The high expression of *pro-resilin* may enable males to jump or pivot wings efficiently.

Interestingly, the open regions near these genes showed similar patterns as gene expression across castes (Fig. 4c), suggesting that their transcriptional regulatory elements are crucial for the differential gene expression. Moreover, we found two genes involved in vision, *retinal homeobox protein Rx1* (*Rx1*) and *glycine receptor subunit alpha-3* (*Glra3*), showing lower levels of both expression and chromatin accessibility in workers, which suggests distinct visual systems across workers and the three other groups. Supporting this, it has been previously reported that ocelli is absent in workers of *Monomorium pharaonis*^54^. In summary, our study provides an important resource of the epigenome and transcriptome of ant brain, which will be of great importance to study the regulatory mechanisms behind caste differentiation in eusocial insects.

## Usage Notes

The RNA-seq data processing pipeline, including data filtering, read mapping and gene expression quantification was run on the Linux operating system (centOS). The optimized parameters are provided in the main text. Differential gene expression (DGE) analysis R source codes used for the downstream data analysis and visualization are provided in Supplementary File 1.

## Supplementary information

Supplementary File 1

## Data Availability

Data processing was performed using open source software. The approach of tools and parameters used were as below. SOAPnuke: https://github.com/BGI-flexlab/SOAPnuke. Version: 1.5.2. Parameters: filter -A 0.5 -M 2 -l 10 -q 0.3 -n 0.05 -Q 2 -d. Cutadapt: https://cutadapt.readthedocs.io/en/stable/. Version: 1.16. Parameters: -m 5 -e 0.10. HISAT2: http://www.ccb.jhu.edu/software/hisat. Version 2.0.1-beta. Parameters: -p 4 –phred33 –sensitive –no-discordant –no-mixed -I 1 -X 1000. featureCounts: http://subread.sourceforge.net/. Version 1.5.3. Parameters: -T 5 -p -t exon -g gene_id. MACS2: https://github.com/taoliu/MACS. Version 2.1.2. Parameters: macs2 callpeak -t input.bam -f BAM -g 259040147 -n name.output -B -q 0.01 --nomodel. Bedtools: https://bedtools.readthedocs.io/en/latest/content/tools/intersect.html. Version: 2.26.0. Parameters: bedtools intersect -a standardpeak.bed -b input.bam -c > output.count. The R code used for calculating the correlation and comparative analysis are available in the supplementary materials.

## References

[1] Libbrecht R (2013). Interplay between insulin signaling, juvenile hormone, and vitellogenin regulates maternal effects on polyphenism in ants. Proc Natl Acad Sci USA.

[2] Nowak M, Tarnita C, Wilson E (2010). The evolution of eusociality. Nature.

[3] Brady SG, Schultz TR, Fisher BL, Ward PS (2006). Evaluating alternative hypotheses for the early evolution and diversification of ants. Proc Natl Acad Sci USA.

[4] Hines HM (2008). Historical biogeography, divergence times, and diversification patterns of bumble bees (Hymenoptera: Apidae: Bombus). Syst. Biol.

[5] Barchuk AR (2007). Molecular determinants of caste differentiation in the highly eusocial honeybee Apis mellifera. BMC Dev Biol.

[6] Berens AJ, Hunt JH, Amy L (2015). Toth. Comparative transcriptomics of convergent evolution: different genes but conserved pathways underlie caste phenotypes across lineages of eusocial insects. Mol Biol Evol.

[7] Qiu B (2018). Towards reconstructing the ancestral brain gene-network regulating caste differentiation in ants. Nat Ecol Evol.

[8] Woodard SH (2011). Genes involved in convergent evolution of eusociality in bees. Proc Natl Acad Sci USA.

[9] Toth AL, Robinson GE (2007). Evo-devo and the evolution of social behavior. Trends Genet.

[10] Toth AL (2010). Brain transcriptomic analysis in paper wasps identifies genes associated with behaviour across social insect lineages. Proc Biol Sci.

[11] Chandra V (2018). Social regulation of insulin signaling and the evolution of eusociality in ants. Science.

[12] Gospocic J (2017). The neuropeptide corazonin controls social behavior and caste identity in ants. Cell.

[13] Johnson BR, Tsutsui ND (2011). Taxonomically restricted genes are associated with the evolution of sociality in the honey bee. PLoS One.

[14] Ferreira PG (2013). Transcriptome analyses of primitively eusocial wasps reveal novel insights into the evolution of sociality and the origin of alternative phenotypes. Genome Biol.

[15] Feldmeyer B, Elsner D, Foitzik S (2014). Gene expression patterns associated with caste and reproductive status in ants: worker‐specific genes are more derived than queen‐specific ones. Mol Ecol.

[16] Mikheyev AS, Linksvayer TA (2015). Genes associated with ant social behavior show distinct transcriptional and evolutionary patterns. Elife.

[17] Simola DF (2013). A chromatin link to caste identity in the carpenter ant Camponotus floridanus. Genome Res.

[18] Simola DF (2016). Epigenetic (re) programming of caste-specific behavior in the ant Camponotus floridanus. Science.

[19] Foret S (2012). DNA methylation dynamics, metabolic fluxes, gene splicing, and alternative phenotypes in honey bees. Proc Natl Acad Sci USA.

[20] Berndt KP, Eichler W (1987). Die Pharaoameise, Monomorium pharaonis (L.)(Hym., Myrmicidae). Mitt. Mus. Nat.kd. Berl., Zool. Reihe.

[21] Wetterer JK (2010). Worldwide spread of the pharaoh ant, Monomorium pharaonis (Hymenoptera: Formicidae). Myrmecological News.

[22] Johnson RA, Overson RP (2017). Population and colony structure and morphometrics in the queen dimorphic little black ant, Monomorium sp. AZ-02, with a review of queen phenotypes in the genus Monomorium. PLoS One.

[23] Picelli S (2013). Smart-seq2 for sensitive full-length transcriptome profiling in single cells. Nat Methods.

[24] Head SR (2014). Library construction for next-generation sequencing: overviews and challenges. Biotechniques.

[25] Davie K (2018). A single-cell transcriptome atlas of the aging Drosophila brain. Cell.

[26] Huang J (2017). A reference human genome dataset of the BGISEQ-500 sequencer. Gigascience.

[27] Andrews, S. *FastQC: A Quality Control Tool for High Throughput Sequence Data*, http://www.bioinformatics.babraham.ac.uk/projects/fastqc/ (2015).

[28] Chen Y (2018). SOAPnuke: a MapReduce acceleration-supported software for integrated quality control and preprocessing of high-throughput sequencing data. Gigascience.

[29] Martin M (2011). Cutadapt removes adapter sequences from high-throughput sequencing reads. EMBnet.journal.

[30] Liu Y (2020). figshare.

[31] Ewels P, Magnusson M, Lundin S, Käller M (2016). MultiQC: summarize analysis results for multiple tools and samples in a single report. Bioinformatics.

[32] Morandin C (2016). Comparative transcriptomics reveals the conserved building blocks involved in parallel evolution of diverse phenotypic traits in ants. Genome Biol.

[33] Kim D, Langmead B, Salzberg SL (2015). HISAT: a fast spliced aligner with low memory requirements. Nat Methods.

[34] Liao Y, Smyth GK, Shi W (2014). featureCounts: an efficient general purpose program for assigning sequence reads to genomic features. Bioinformatics.

[35] Love MI, Huber W, Anders S (2014). Moderated estimation of fold change and dispersion for RNA-seq data with DESeq2. Genome Biol.

[36] Koh PW (2016). An atlas of transcriptional, chromatin accessibility, and surface marker changes in human mesoderm development. Sci Data.

[37] Zhang Y (2008). Model-based analysis of ChIP-Seq (MACS). Genome Biol.

[38] Li Q, Brown, James. B, Huang H, Bickel PJ (2011). Measuring reproducibility of high-throughput experiments. Ann. Appl. Stat.

[39] Quinlan AR, Hall IM (2010). BEDTools: a flexible suite of utilities for comparing genomic features. Bioinformatics.

[40] Schug J (2005). Promoter features related to tissue specificity as measured by Shannon entropy. Genome Biol..

[41] (2019). Gene Expression Omnibus.

[42] CNGB (2019). Nucleotide Sequence Archive.

[43] Ou J (2018). ATACseqQC: a Bioconductor package for post-alignment quality assessment of ATAC-seq data. BMC Genomics.

[44] Buenrostro JD, Giresi PG, Zaba LC, Chang HY, Greenleaf WJ (2013). Transposition of native chromatin for fast and sensitive epigenomic profiling of open chromatin, DNA-binding proteins and nucleosome position. Nat Methods.

[45] Claeys I (2002). Insulin-related peptides and their conserved signal transduction pathway. Peptides.

[46] Hetru C, Li KW, Bulet P, Lagueux M, Hoffmann JA (1991). Isolation and structural characterization of an insulin‐related molecule, a predominant neuropeptide from Locusta migratoria. Eur J Biochem.

[47] Wu Q, Brown MR (2006). Signaling and function of insulin-like peptides in insects. Annu Rev Entomol.

[48] Lagueux M, Lwoff L, Meister M, Goltzené F, Hoffmann JA (1990). cDNAs from neurosecretory cells of brains of Locusta migratoria (Insecta, Orthoptera) encoding a novel member of the superfamily of insulins. Eur J Biochem.

[49] Tufail M, Nagaba Y, Elgendy AM, Takeda M (2014). Regulation of vitellogenin genes in insects. Entomological Science.

[50] Corona, M. *et al*. Vitellogenin underwent subfunctionalization to acquire caste and behavioral specific expression in the harvester ant Pogonomyrmex barbatus. *PLoS Genet* **9** (2013). 10.1371/journal.pgen.1003730 PMC3744404 23966882

[51] Ukmar-Godec T (2019). Lysine/RNA-interactions drive and regulate biomolecular condensation. Nat Commun.

[52] Warner MR, Qiu L, Holmes MJ, Mikheyev AS, Linksvayer TA (2019). Convergent eusocial evolution is based on a shared reproductive groundplan plus lineage-specific plastic genes. Nat Commun.

[53] Qin G, Hu X, Cebe P, Kaplan DL (2012). Mechanism of resilin elasticity. Nat Commun.

[54] Narendra A, Ramirez-Esquivel F, Ribi WA (2016). Compound eye and ocellar structure for walking and flying modes of locomotion in the Australian ant, Camponotus consobrinus. Sci Rep.

